# The Evolution of Leaf Function during Development Is Reflected in Profound Changes in the Metabolic Composition of the Vacuole

**DOI:** 10.3390/metabo11120848

**Published:** 2021-12-06

**Authors:** Alice Destailleur, Théo Poucet, Cécile Cabasson, Ana Paula Alonso, Jean-Christophe Cocuron, Romain Larbat, Gilles Vercambre, Sophie Colombié, Pierre Petriacq, Marie Hélène Andrieu, Bertrand Beauvoit, Yves Gibon, Martine Dieuaide-Noubhani

**Affiliations:** 1 UMR Biologie du Fruit et Pathologie, Université de Bordeaux, INRAE, F-33140 Villenave d’Ornon, France; alice.destailleur@u-bordeaux.fr (A.D.); t.poucet@outlook.fr (T.P.); cecile.cabasson@u-bordeaux.fr (C.C.); sophie.colombie@inrae.fr (S.C.); pierre.petriacq@inrae.fr (P.P.); marie-helene.andrieu@inrae.fr (M.H.A.); bertrand.beauvoit@inrae.fr (B.B.); yves.gibon@inrae.fr (Y.G.); 2 Bordeaux Metabolome, MetaboHUB, PHENOME-EMPHASIS, F-33140 Villenave d’Ornon, France; 3 Department of Biological Sciences, BioDiscovery Institute, University of North Texas, Denton, TX 76203, USA; Anapaula.Alonso@unt.edu; 4 BioAnalytical Facility, University of North Texas, Denton, TX 76203, USA; Jeanchristophe.Cocuron@unt.edu; 5 LAE, Université de Lorraine, INRAE, F-54000 Nancy, France; romain.larbat@univ-lorraine.fr; 6 Plants and Cropping Systems in Horticulture, INRAE, F-84914 Avignon, France; gilles.vercambre@inrae.fr

**Keywords:** subcellular metabolome, tomato leaf, vacuole, non-aqueous fractionation

## Abstract

During its development, the leaf undergoes profound metabolic changes to ensure, among other things, its growth. The subcellular metabolome of tomato leaves was studied at four stages of leaf development, with a particular emphasis on the composition of the vacuole, a major actor of cell growth. For this, leaves were collected at different positions of the plant, corresponding to different developmental stages. Coupling cytology approaches to non-aqueous cell fractionation allowed to estimate the subcellular concentrations of major compounds in the leaves. The results showed major changes in the composition of the vacuole across leaf development. Thus, sucrose underwent a strong allocation, being mostly located in the vacuole at the beginning of development and in the cytosol at maturity. Furthermore, these analyses revealed that the vacuole, rather rich in secondary metabolites and sugars in the growth phases, accumulated organic acids thereafter. This result suggests that the maintenance of the osmolarity of the vacuole of mature leaves would largely involve inorganic molecules.

## 1. Introduction

The production and transport of sugars and amino acids, from the source leaves to the sink organs, play a major role in plant growth and performance [1]. In this context, the developing leaf is particularly interesting to study, because it goes from the status of a sink organ to that of a source. Thus, the youngest leaves, although photosynthetically active, must import assimilated compounds to allow their growth [2] while mature leaves ensure the carbon supply to the sink organs, such as roots, flowers, fruits, buds and young leaves. Metabolic and/or transcriptomic changes, which occur during source-sink transition have been studied in several species, such as tobacco [3], quaking aspen [4], maize [5], tomato [6] and rice [7].

Leaf growth and development are complex processes involving both metabolic and physical changes. For instance, the water potential, higher in the soil than in the plant, and transpiration through the stomata during the day play an important role ensuring a flow of water towards the leaf cells. Cell growth is then allowed by movements of water and solutes into the vacuole which ensure cell turgor pressure [8,9].

Vacuoles are single membrane organelles present in all plant cells. They are classified in (i) lytic vacuoles, enriched in lytic enzymes such as hydrolases, proteinases and defense proteins, usually found in vegetative tissues and (ii) storage vacuoles, less acidic, that accumulate proteins, and more specific to seeds [10,11]. Vacuoles contain a wide range of organic (mainly soluble sugars, amino acids, organic acids and secondary metabolites such as flavonoids, isoprenoids) and inorganic (mineral ions) compounds, and can also store phosphorylated compounds [12], or accumulate toxic pollutants such as cadmium or arsenic when plants are grown on contaminated soils [13]. Vacuoles share most of these compounds with the cytosol, but the knowledge of their subcellular distribution is still fragmentary. This is an important issue for plant metabolomics since the vacuole occupies a significant part of the cell volume, ranging from 60% in barley leaf [14] to 80% in tomato fruits [15], and neglecting this compartment could lead to misinterpretation of cell metabolism. To overcome the fragility of the vacuole, it can be isolated from protoplasts [16], but a major disadvantage of such a fractionation method performed in aqueous phase, is that it does not guarantee the integrity of the metabolite contents due to possible diffusion leakage. Moreover, the purification steps generally lead to losses, in such a way that a non-representative subgroup of the organelle under study could be selected. One alternative is the non-aqueous fractionation (NAF), which was initially developed to study ATP/ADP ratios in cytosol and mitochondria of animal tissues [17]. It consists in breaking frozen cells into particles that are then lyophilized. After suspension in an anhydrous tetrachloroethylene/heptane mixture, the particles are separated by centrifugation on a gradient composed of these same organic solvents. Then, the fractions of the gradient are characterized using specific markers of subcellular compartments such as enzymes activities or metabolites, and a statistical analysis allows to calculate the relative abundance of assayed metabolites in each characterized compartment. Applied to plant tissues, NAF enables the cell to be described by a three-compartment model involving vacuole, chloroplast and cytosol. Mitochondria are generally not sufficiently resolved from cytosol, and the term “cytosol” refers to “mitochondria plus cytosol” [12,18,19]. This approach allowed to characterize the subcellular distribution of the major metabolites in spinach [19,20], Arabidopsis [18], maize or soybean [21] leaves, but also in rose petals [22], barley seeds [23] and potato tuber [12] and leaves [24]. In a few cases, subcellular concentrations of metabolites were also estimated by coupling the NAF with a cytological analysis [12,14,20]. More specifically, the subcellular concentrations of thehalose-6-phosphate were studied in Arabidopsis leaves in relation with the regulation of starch breakdown [25]. Associated with isotope labeling, NAF allowed the estimation of fluxes in Calvin-Benson–Bassham (CBB) cycle reactions in Arabidopsis leaves [26]. More recently, it has been used to characterize changes in the distribution of metabolites and inorganic ions in developing apple fruit [27].

The characterization of leaf metabolism at different phenological stages allowed a better dissection and understanding of numerous processes, e.g., the sink-source transition and leaf senescence [3,28,29] or the response of tomato leaf to different nitrogen sources [30]. However, there is scarce information about the influence that development may exert on the subcellular repartition and concentration of main metabolites of leaf cells.

In this work, we applied NAF procedure to tomato leaves sampled at different positions on the plant. To determine the subcellular concentrations of metabolites, the volumes of these compartments were measured according to the leaf position on the tomato plant. The analysis of metabolites distribution in sub-cellular compartments was discussed according to the leaf stage.

## 2. Results

### 2.1. Leaf Growth and Changes in Cellular and Subcellular Volumes

**Figure 1:**
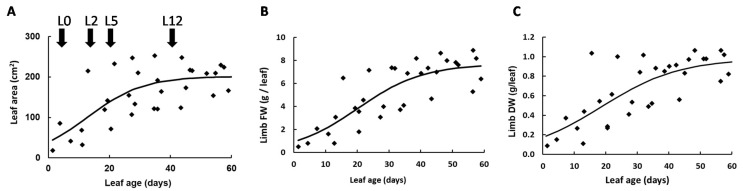
Evolution of leaf area (**A**), limb fresh weight (**B**) and dry weight (**C**) according to the age of tomato leaves. Leaves were harvested throughout the experiment and the curves obtained were used to determine the age of the leaves L0, L2, L5 and L12.

This study was designed to investigate metabolic composition in tomato leaves, according to their position on the plant. Since metabolism fluctuates between day and night [31], leaves were harvested at the end of the night (EN) and at the end of the day (ED). The study was restricted to the limb owing to high sucrose and amino acid concentrations in veins that would impact data consistency. The first harvested leaf, named L0, was collected near the top of the plant and was 18 to 22 cm long. The other leaves were collected below L0, at positions −2 (L2), −5 (L5) and −12 (L12). To estimate their age, five plants were harvested and the length, area, fresh and dry weights (FW and DW) were measured for each leaf (Figure 1). Based on a simple growth model and on the phyllochron [32], L0, L2, L5 and L12 were estimated to be 12, 18, 27 and 47 days old, respectively. As shown in Figure 1A, the leaf area increased significantly between L0 to L5, indicating that these leaves were still growing, and then, reached a plateau for L12. Leaf FW (Figure 1B) and DW (Figure 1C) presented a similar evolution, except that they increased slightly between L5 and L12, which may reflect an increase in leaf thickness.

**Figure 2:**
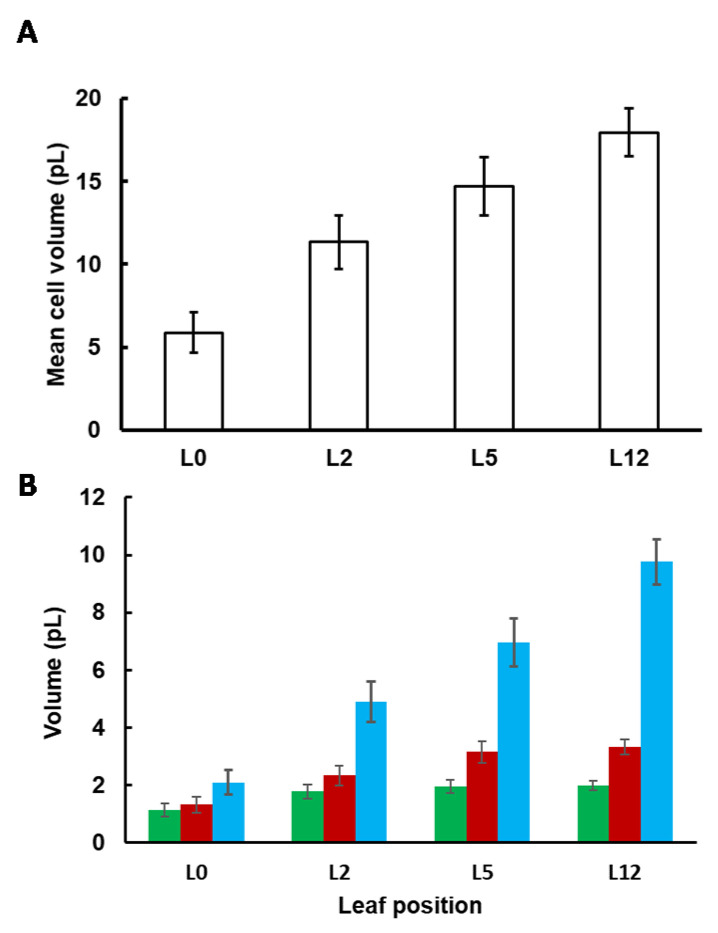
Cell (**A**) and subcellular (**B**) volumes of tomato leaves according to their position on the plant. To calculate the absolute volume of the chloroplast (■), the cytosol (■) and the vacuole (■), the mean cell volume was multiplied by the fractional volume of each compartment. Values were corrected as described in [10,16] to take into account the shrinkage of the leaf during the fixation process. Values are the mean (*n* = 3 leaves) ± SE.

Cell and subcellular volumes (Figure 2) were estimated as described in the Section 4. The cell volume continuously increased, even between L5 and L12, from 5.9 ± 1.2 to 18.0 ± 1.4 pL. This increase resulted from an enlargement of the vacuole and to a lesser extent of the cytosol, whereas the volume occupied by the chloroplasts remained almost constant (Figure 2B). Interestingly, the relative volume of the vacuole increased from 35% to 55%, at the expense of the chloroplasts whose relative volume shrunk from 19% to 11% of the cell volume (Figure S1). On the other hand, the relative volume of the cytosol was stable along development, occupying around 20% of the cell volume.

### 2.2. The Young Leaf Contains More Polyphenols and Phosphorylated Compounds but Less Amino Acids and Organic Acids

**Figure 3:**
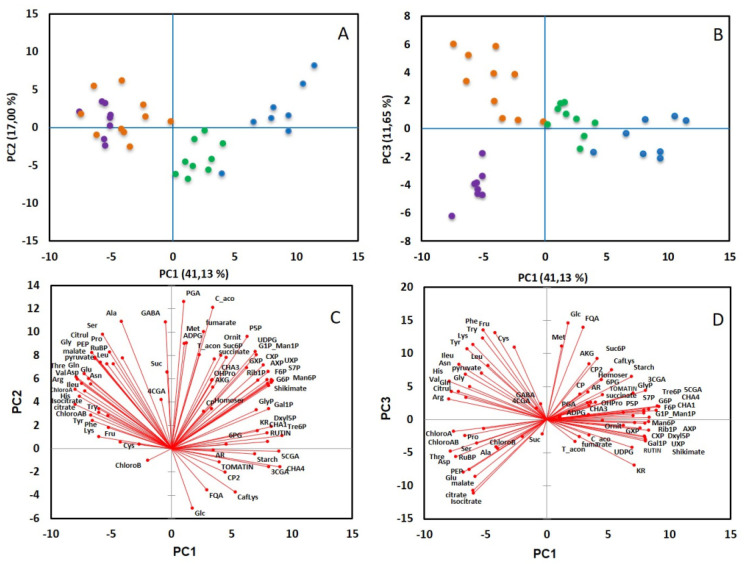
Principal component analysis of metabolites measured in tomato leaves L0 (●), L2 (●), L5 (●) and L12 (●). PCA was performed from the correlation matrix generated with a total of 65 metabolites, with 3 to 5 replicates per leaf position and period of the day. (**A**,**B**) represents the scores plots, (**C**,**D**) the loadings plots according to axes 1 and 2 (**A**,**C**) or 1 and 3 (**B**,**D**).

Fifty-six compounds were measured in tomato leaf using LC-MS/MS and enzymatic methods (Table S1). To visualize the data, a principal component analysis (PCA) was performed with diel time (end of day or end of night) or leaf position (L0 to L12) as factors. Considering leaf position, the first three components (PC1, PC2 and PC3), which explain 41, 17 and 11% of the total variance, respectively (Figure 3), were required to discriminate the four positions of leaves: PC1 and PC2 separated the samples from L0 to L5, whereas PC3 differentiated samples from leaves L5 and L12. The first two axes separate most of the leaves according to their growth. Considering leaves L2 to L12, those harvested at the end of the night were distinguished from those harvested at the end of the day (Figure S2). The discrimination of these two groups can be explained by the levels of sucrose, starch and phosphorylated intermediates of the CBB cycle that were always lower at the end of the night. The compounds showing the most dramatic changes were starch (reduced by 30 to 50% at the end of the night, according to the developmental stage), xylulose 5-P (decreased by 50 to 75%) and ribulose-1,5-bisphosphate (RuBP) that was decreased by a factor 3 in leaves L2 and L5 and a factor 26 in leaf L12 (Figure S3). It should be noticed that ornithine content was significantly decreased at the end of the night (Table S1). This result agrees with the fact that ornithine is a precursor for the polyamines, such as putrescine, whose synthesis is affected by darkness and regulated by the diurnal cycle [33].

In the tomato leaf, the major amino acids, representing 90% of the total amino acids, were Asn, Asp, γ-aminobutyric acid, Glu, Gln, Phe and Ser (Table S1). Leaf growth was accompanied by an increase in amino- and organic acid contents expressed on a dry weight basis (Figure S3). When comparing L5 and L12 leaves, most of amino acid contents were similar (Gln, Asn, Gly, Arg, Val) or decreased (Met, Phe), whereas Glu and Asp increased. Besides, PEP and organic acids (malate, citrate and isocitrate) went on increasing. Free sugars were mainly glucose (Glc), fructose (Fru) and sucrose. Sucrose did not vary significantly according to leaf position. In L0 and L2, glucose and fructose contents were similar, whereas glucose content decreased gradually in older leaves, leading to an increase in the Fru-to-Glc ratio from 1.2 ± 0.2 to 3.0 ± 0.4 (Figure S9). Owing to sucrose stability, the hexose-to-sucrose ratio rose between L0 and L5 from 6.0 ± 2.8 to 9.2 ± 1.4 at the end of the day, 9.1 ± 2.8 to 15.5 ± 1.4 at the end of the night, and then decreased close to the value measured in L0. Interestingly, nitrate content progressively decreased from the bottom to the top of the plant. The highest nitrate concentrations were found in the oldest leaves, which accumulate amino acids (L5 and L12), especially Gln and Asn. It suggests greater nitrogen assimilation activity in these leaves [34] compared to the young ones (L0 and L2) [30]. Concentrations of malate reached a maximum in L5 and L12, and were also higher at the end of the day (Figure S3), which is in agreement with the involvement of malate in buffering OH^−^ during light-dependent assimilation of nitrate [35].

Along leaf development, secondary metabolites such as caffeoyl hexaric acid isomers (CHA1, 3 and 4), chlorogenic acids (CGA) and kaempferol rutinoside (KR) decreased. RuBP and starch contents decreased significantly between L5 and L12 (Table S1 and Figure S3), probably due to a reduction of the photosynthetic activity caused by crop shading [36]. Photosynthetic assimilates of young leaves are mainly used for their own growth, whereas in mature leaves, because maintenance costs are much lower than growth costs, most of the carbon fixed by photosynthesis is exported to the sinks. As previously observed in tomato [31], starch content was only slightly affected by the diel cycle compared to plants such as Arabidopsis in which only 5 to 10% remains at the end of the night (Figure S3) [37]. On the other hand, a significant decrease in starch content was observed along leaf development, contrasting with chlorophyll content, which remained stable (Table S1). These observations suggest that starch could be used more as a carbon pool that can be remobilized for leaf growth, rather than a transient reserve to support the nocturnal needs of the whole plant.

### 2.3. Non-Aqueous Fractionation and Subcellular Distribution of Metabolites

**Figure 4:**
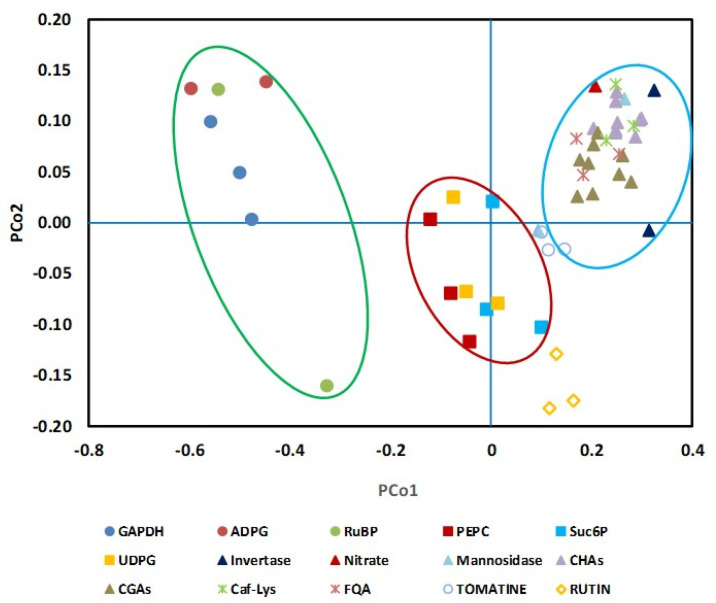
Validation of new subcellular markers in tomato leaf. On the basis of their repartition in the NAF gradients performed with leaf L5, the Manhattan distance was calculated between well-known markers (PEPC, NADP-GAPDH and α-mannosidase) and selected metabolites or enzymes (Invertase, Nitrate, ADPG: ADP-Glucose; RuBP: ribulose-1,5-bisphosphate Suc6P: sucrose-6-phosphate; UDPG: UDP-glucose; CHAs: caffeoyl hexaric acid isomers: CHA1, CHA3 and CHA4; CGAs: chlorogenic acid isomers: 3CGA, 4CGA and 5CGA; Caf-Lys: caffeoyl-lysine; FQA: Feruloyl quinate, Tomatine and Rutin). A multidimensional scaling (CMD) was then performed for the three independent gradients to transform the data into a two-dimensional dataset. The green, red and blue ellipses group metabolites or enzymes associated to chloroplast, cytosol and vacuole, respectively.

NAF was performed with leaves harvested at the four developmental stages and, because of the minor impact of the diel cycle, only at the end of the night. Fractions obtained from the gradients were first characterized by measuring markers of the subcellular compartments, i.e., the enzymes that are classically monitored but also some metabolites, as distribution of the metabolites between the subcellular compartments using a linear regression model optimized by the least square method. Depending on the developmental stage, markers were sometimes not detectable or below the limit of detection. For instance, acid invertase activity and nitrate could only be measured in L12 and L5 samples. As nitrate is considered to be almost completely vacuolar in leaves [14,20], it was used as a vacuole marker in Arabidopsis [18]. Figure 4 exemplifies the distribution of nitrate, acid invertase (when detectable) and phenylpropanoids in non-aqueous fractionations performed on L5 samples.

According to our data, most of the phenylpropanoids co-located with nitrate and acid invertase, indicating they were mainly vacuolar. Only rutin presented a particular pattern that could not be explained by the tri-compartmental model. As shown in Figure S4, the caffeoyl hexaric acid isomers (CHA1, 3 and 4) and the chlorogenic acids had also a vacuolar localization when considering the leaves in positions L0, L2 and L12. Our data also showed that phosphoenolpyruvate carboxylase (PEPC), sucrose-6P and UDP-Glc on one hand, and RuBP, ADP-Glc, chloroplyll and GAPDH on the other hand, could be used as markers for cytosol and plastid, respectively (Figure 4 and Figure S4).

Surprisingly, in leaves L0 and L2, chlorophyll, RuBP and ADP-Glc were very close to each other, but separated from NADP-dependent glyceraldehyde dehydrogenase (NADP-GAPDH) activity (Figure S4). This discrepancy was probably due to interferences in the NADP-GAPDH assay in some fractions. Chloroplasts are classically found at the top of the gradient due to their relatively low density, the vacuole in a pellet at the bottom of the tube, whereas cytosol markers are distributed along the gradient. This classical pattern was observed with L5 and L12. Considering the younger leaves (L0 and L2, Figure S4), the distribution of markers in the gradient varied considerably according to the sample, and we did not observe a clear separation of chloroplast and cytosol compartments. For the oldest leaves (L12), the repeatability of the fractionation increased and the separation of the three compartments was optimal for leaf in the intermediate stage (L5).

**Figure 5:**
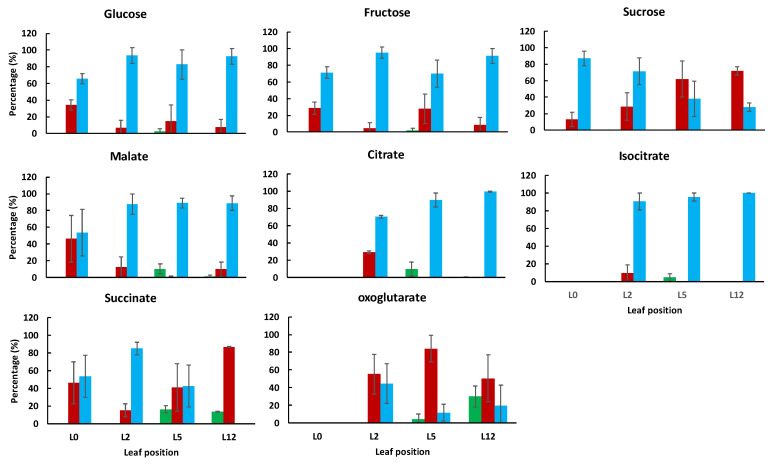
Subcellular distribution of the major sugars and organic acids within the chloroplast (green), cytosol (red) or vacuole (blue) in tomato leaves harvested at 4 positions on the plant. For each metabolite, values are expressed as percentages and are the mean (*n* = 3) ± SD, except for isocitrate in leaf 12 (only one value).

The subcellular compartmentation of assayed metabolites was then calculated using the distribution of markers, from three independent samples. The distribution of free sugars (glucose, fructose and sucrose) and organic acids (citrate, isocitrate, malate, fumarate and succinate), usually considered as mainly located within the vacuole and the cytosol [20,21,26], was studied among the four stages (Figure 5) using a tri-compartmented model for the oldest leaf samples (L5 and L12) and a bi-compartmented model for the youngest leaf samples (L0 and L2). It should be noted that for L5 and L12, the distribution of all sugars, organic acids and secondary metabolites using a bi-compartmented model was almost similar to that obtained with a tri-compartmented model (Figure S5), thus strengthening that these metabolites are not, or only marginally, located in the plastid.

Glucose and fructose were predominantly vacuolar, independently of the leaf developmental stage. Sucrose, which was mainly vacuolar in the youngest leaves, became progressively cytosolic as they grew. The cytosolic localization of sucrose has previously been observed in fully developed leaves of Arabidopsis, spinach and barley [14,18,20]. In tomato leaves, the vacuolar localization of sucrose could reflect the status of the youngest leaf, in which acidic invertase activity is low (Table S1, and Figure S6). In agreement with results obtained in literature [26], citrate, malate and isocitrate were predominantly located into the vacuole, with values comprised between 87 and 100% in leaf samples L5 and L12. The highest cytosolic percentage of citrate was found in L2. Succinate was clearly associated with the vacuolar fraction in L2 and the cytosolic fraction in L12. For the leaf L5, its subcellular distribution was determined with a relatively large error which could be a consequence of the transition from the vacuolar to the cytosolic localization. Fumarate distribution was not reported because it was not well explained with the tri-compartmented model.

A significant proportion of amino acids and phosphorylated compounds was expected to localize within the chloroplast. Their distribution was analyzed in L5 and L12, only when the separation of the three compartments was consistent (Figures S7 and S8). According to our data, Asn in L5 and L12, and GABA in L12 were predominantly vacuolar (60 to 80%). All other amino acids were found in the three subcellular compartments, with significant amount of Asp, Glu and Phe in the chloroplast (35 to 40%). Fifteen phosphorylated compounds were analyzed (Table S2). Among them, xylulose-5-P, erythrose-4-P and trehalose-6-P were considered as undetermined and were not further analyzed. As expected, the phosphorylated compounds were predominantly distributed between the cytosol and the chloroplast (Figure S8). The intermediates upper part of the glycolysis, namely glucose-6-phosphate, fructose-6-phosphate and fructose-1,6-bisphosphate, were predominantly localized in the cytosol (60–80%), whereas a major part of phosphoenolpyruvate and pyruvate was in the chloroplast. We did not succeed in the separation of 2-phosphoglyceric acid from 3-phosphoglyceric acid, which were also found equally distributed between cytosol and plastid, in agreement with the dual localization of the glycolytic enzymes. An intermediate of the oxidative pentose phosphate pathway (PPP), 6-phosphogluconate, was predominantly present in the chloroplast, whereas sedoheptulose-7-phosphate and pentose-5-phosphate were similarly distributed between the cytosol and the chloroplast, thus suggesting that the non-oxidative part of the PPP could also be active within the cytosol. Small amounts of fructose-6-phosphate and mannose-6-phosphate were also found in the vacuole. Phosphorylated sugars have previously been found in the vacuole of potato tubers, which contained 34% of the glucose-1-phosphate [12]. Unfortunately, the methodology used in our study was not able to separate glucose-1-phosphate from mannose-1-phosphate.

### 2.4. Subcellular Concentrations of Sugars and Organic Acids Change According to Leaf Developmental Stage

**Figure 6:**
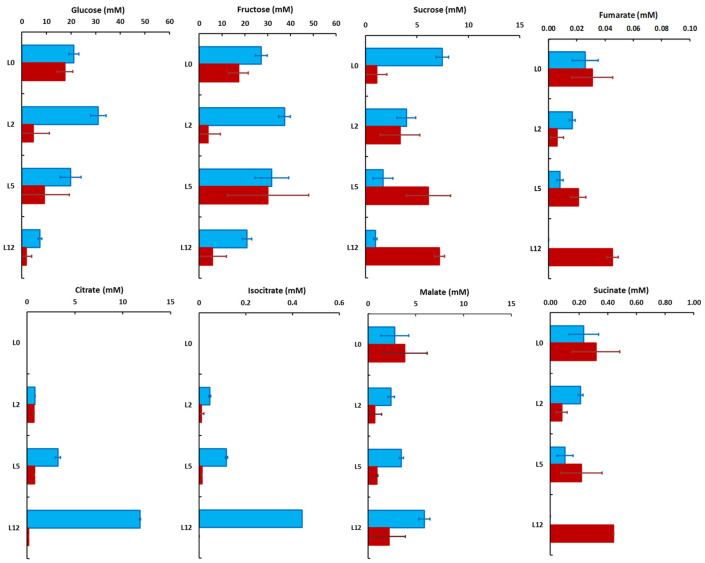
Vacuolar (blue) and cytosolic (red) concentrations of the major sugars and organic acids in tomato leaves harvested at four positions on the plant. The values were calculated from the mean distribution, and the errors reflect the errors on the determination of the subcellular distributions. Values are the mean (*n* = 3) ± SD, except for isocitrate in leaf 12 (only one value).

The concentrations of the metabolites within each compartment were estimated using the subcellular distribution, the total content and the subcellular volume (Table S2). The variations in cytosolic and vacuolar concentrations of hexoses largely reflect those of their total content as their distribution did not vary significantly along leaf development (Figure 6).

In leaves L0 to L5, the cytosolic and vacuolar concentrations of glucose ranged from about 20 to 30 mM in the vacuole and about 5 to 18 mM in the cytosol, and then decreased respectively to 7.3 ± 0.7 and 1.8 ± 2.2 mM in L12. The vacuolar concentration of fructose remained high throughout the development, ranging from 21 to 37 mM, whereas the cytosolic values fluctuated more, between 5 and 30 mM (Figure 6). Interestingly, the vacuolar and cytosolic concentrations of sucrose were more affected, decreasing from 7.5 ± 0.6 to 0.9 ± 0.2 mM in the vacuole while increasing from 1.1 ± 1.0 to 7.2 ± 0.5 mM in the cytosol between leaves L0 and L12 (Figure 6). Accordingly, the hexose/sucrose ratio was drastically decreased in the cytosol whereas it increased from about 6 to 30 into the vacuole (Figure S9). In fully developed tomato leaves, the photosynthetic products are mostly exported during the day, thus explaining the small differences in sugars between the end of the day and the end of the night [31]. The augmentation of UDP-glucose pyrophosphorylase activity between leaves L2 and L5 (Figure S6) is in good agreement with the increase in sucrose synthesis and cytosolic content in the leaf during the day.

Malate was the major organic acid in L0, with similar cytosolic and vacuolar concentrations (3.9 ± 2.3 and 2.8 ± 1.5 mM, respectively) whereas malate became mostly vacuolar reaching a concentration of 5.9 ± 2.2 mM in L12. Meanwhile, in L5 and L12, citrate progressively became the major organic acid. It was mostly vacuolar with a concentration increasing from 0.84 ± 0.02 mM in L2 to 11.81 ± 0.06 mM in L12 (Figure 6). On the other hand, the cytosolic concentrations of malate and citrate remained stable, close to 2.5 and 0.8 mM, respectively. Isocitrate presented the same pattern as citrate, except that it was about 30 times less concentrated (Figure 6). Vacuolar succinate concentration decreased, whereas the cytosolic concentrations slightly increased, probably due to a reallocation into the cytosol (Figure 6).

Because amino acids were found in the three subcellular compartments, it was not possible to calculate their cytosolic concentration at the four developmental stages. It was thus decided to consider the cytosol and the chloroplasts as a unique compartment, and to estimate the vacuolar and the cytoplasmic concentrations (Figure S10). For most of the amino acids, their concentrations remained relatively stable and similar between the cytoplasm and the vacuole. One exception was asparagine, the major amino acid in tomato leaf, which accumulated in the oldest leaves, especially in the vacuole of L5.

## 3. Discussion

### 3.1. Non-Aqueous Fractionation Efficiency Depends on Leaf Developmental Stage

The aim of this work was to apply NAF to tomato leaves harvested at different positions on the plant, corresponding to different developmental stages, to evaluate compositional changes in relation to leaf age and growth. Although NAF protocols are quite well documented, successful fractionation depends on the ability to generate and separate cell particles on a density gradient and on the availability of measurable markers for each compartment. Classically used as vacuolar marker, α-mannosidase activity was very low in tomato leaf, leading to poor determination of the vacuole localization along the gradient. Moreover, measurements of the plastid marker NADP-GAPDH were clearly biased in fractions from the youngest leaves. Such difficulty has been reported for apple fruit fractionation [27] in which NADP-GAPDH was not detectable and was consequently replaced by starch as a plastid marker. In Arabidopsis leaf, metabolites have also been used as additional markers [18], such as starch and digalactosyldiacylglycerol for the chloroplast and nitrate or flavonoids for the vacuole. In this work on tomato leaf, we identified new metabolites and enzymes as markers, namely (1) UDP-glucose and sucrose-6-phosphate presenting the same profile as PEPC were chosen as complementary markers for the cytosol, (2) ADP-glucose and RuBP for the chloroplast, and (3) nitrate, phenolic acid esters (chlorogenic and caffeoyl hexaric acids) and acid invertase for the vacuole. The phenylpropanoid biosynthesis takes part mostly in the cytosol through cytosolic and ER associated enzymes [38,39], although metabolically active phenylpropanoïd related enzymes were also found in the vacuole [40]. These metabolites would be then stored in the vacuole, usually in a conjugated form after being imported either through specific ligandin transporters (TT19 in Arabidopsis, [41]) or through autophagy-related direct import from ER/cytoplasm into the vacuole [42]. Vacuolar localization of flavonoids has been reported for vacuoles isolated from barley mesophyll protoplasts [16] and recently confirmed with non-aqueous fractionation in apple fruit [27]. Surprisingly, the major flavonoid in tomato, rutin was not only associated with the vacuole, but was also largely distributed in the cytosol. Similar results were obtained with tomatine, a major glycoalkaloid in young fruits. Their extra-vacuolar location is probably linked to their secretion into the apoplast [43], a compartment that is neglected in the tri-compartmental model. The interest in multiplying markers is therefore to avoid the bias due to the low abundance of enzymes and/or to their inactivation by freeze-drying and/or the use of organic solvents. The accuracy becomes even greater when these extra-markers are measured by high-sensitivity MS.

In this work, the results obtained with fractionations performed on leaves L5 and L12 are consistent with the literature data. Indeed, marker distributions indicate that chloroplasts were mainly on the top of the gradient, corresponding to the less dense fraction, while the densest fraction contained 50 to 60% of vacuoles. Finally, the cytosol was distributed within the whole gradient. Moreover, the high reproducibility of the separation is in agreement with result on Arabidopsis leaf [18]. On the contrary, the separation between the cytosol and the plastids was not feasible in the youngest leaf samples. The failure to discriminate subcellular compartments has previously been reported in barley seeds, in which vacuole markers were not separated from cytosolic ones. The authors hypothesized that the vacuoles were too small to be separated from the other subcellular compartments, or that their composition was such that their density was close to that of the cytosol [23]. In the youngest leaves, the volumes of the three subcellular compartments were similar, thus ruling out the hypothesis that plastid enriched particles were not detectable. Tiessen and co-authors [23] underlined that particles have to be as small as possible to reduce the risk of including material from all organelles. Although it cannot exclude that, in the youngest leaf, the chloroplast density is close to that of the cytosol, another plausible explanation could be that in these leaves, the cell volume is low and the particle size not small enough to discriminate a plastid enriched fraction.

### 3.2. Composition and Storage Capacity of the Vacuole Change throughout Leaf Development

Vacuole plays a major role in cytosolic homeostasis and cell growth by accumulating sugars, organic acids, amino acids and ions but also secondary metabolites and toxic compounds [44]. NAF has been used to estimate the subcellular repartition of sugars and organic acids within plant cells [18,21,22], and in a few cases, their subcellular concentrations [12,23,24,25,26]. All these studies revealed that sugars and organic acids are mostly located in the vacuole and the cytosol, and marginally in the plastid (less than 10%). In tomato leaf, total sucrose did not vary significantly between L0 and L12. In the oldest leaves, sucrose was predominantly (60 to 80%) localized into the cytosol, as previously observed in spinach or barley leaves harvested at the end of a dark period [14,19]. However, fractionation revealed that sucrose was mainly vacuolar in the youngest leaf and became progressively cytosolic as the leaf grew. Important sucrose reallocation is known to occur in spinach leaf, where the percentage of vacuolar sucrose changes during a light/dark cycle, decreasing from 80% at the end of the light period to 32% at the end of the dark one [19]. While the compartmentalization of sucrose changed significantly during leaf development, glucose and fructose distribution did not change markedly, remaining predominantly vacuolar, providing new insight into leaf sugar metabolism. Indeed, at the cell or tissue level, the ratio between hexoses and sucrose only changed marginally during leaf development, but it changed dramatically at the subcellular level, decreasing from 31.6 to 1.1 in the cytosol and increasing from 6.5 to 29.6 in the vacuole (Figure S9). The concentration of a given metabolite results from the balance between its synthesis and degradation. In the case of mature leaves, the strong increase in cytosolic sucrose could be interpreted as favoring its passive phloem uploading [30], and thus, its export to sink tissues like the very young leaves. In that view, the strong decrease in vacuolar sucrose concentrations may result from a reprogramming of sucrose metabolism leading to higher activity of the vacuolar invertase (Figure S6), possibly coupled with lower sucrose import into the vacuole, which would explain the increased hexose-to-sucrose ratio found in that compartment. Identifying the mechanisms involved in this shift could provide a better understanding of how source-sink regulation operates within leaves and lead to new strategies aimed at optimizing the allocation of resources in crops.

In contrast to sucrose, the subcellular compartmentation of organic acids did not change significantly during leaf development. In the case of citrate and isocitrate, their concentrations increased in the vacuole as a consequence of their accumulation in the tissue. As expected [16], malate was also found mainly in the vacuole. However, it can be noticed that the cytosolic and vacuolar concentrations were of the same order of magnitude.

The composition of the vacuole was compared between the different developmental stages by summing the concentrations of the measured metabolites. As shown in Figure S11, the total concentration of assimilated compounds decreased from L0 to L12. This decrease results, in part, from a diluting effect due to the vacuole expansion. In growing leaves (L0 and L2), the major metabolites were glucose, fructose and secondary metabolites that represent 83 to 88% of the metabolites measured in the vacuole. In mature leaves, free sugars and secondary metabolites decreased whereas organic acids increased. Total secondary metabolites, the major ones being rutin and tomatine, decreased from about 28 mM in L0 to 3 mM in L12 (Figure S11), thus constituting the main drivers in the decrease of total composition. One explanation could be that since these compounds are involved in plant defense, they are more concentrated in the youngest leaves than in fully expanded leaves [45]. The reduction in total metabolite concentration and the increase in organic acids suggest that inorganic ions, especially cations, should accumulate to satisfy electroneutrality and to maintain total osmolarity. This result agrees with recently published work showing that osmolarity increased in tomato leaf from the top to the bottom of the plant and that the content in ions, mainly sulfate, Ca^2+^ and Mg^2+^, was higher in fully expanded leaves than in younger ones [30]. This result suggests that in fully developed leaves, vacuole osmolarity is mostly maintained with inorganic compounds rather than photoassimilated compounds. Besides, the oldest leaves are closest to the roots and therefore probably the first receiving mineral nutrition.

## 4. Materials and Methods

### 4.1. Plants Culture and Leaf Harvesting

Experiments were performed with *Solanum lycopersicum* L. cultivar Moneymaker, as described in [46]. Leaves were harvested at the end of October, at the end of the night (EN, 2 h 30 min–1 h 50 min before sunrise) and at the end of the day (ED, 30 to 70 min before sunset). Four leaves were harvested per sample and development stage on each plant, and the limb was rapidly cut and frozen. The first harvested leaf, named L0, was collected near the top of the plant and was 18 to 22 cm long. The other leaves were collected below L0, at the positions −2 (L2), −5 (L5) and −12 (L12). The age of the leaves was deduced from growth curves established throughout the experiment.

### 4.2. Non-Aqueous Fractionation

Leaf samples were ground into a fine powder using a ball mill (Retsch, 2 min, 30 Hz) and then freeze-dried for 4 days. Sample temperature was then increased to room temperature before opening the freeze-drier and samples were stored at −20 °C in a tube surrounded by silica gel.

For non-aqueous fractionation, the protocol was similar to that described by [18]. Briefly, 200 mg of dried powder were resuspended in 20 mL 66:34 (*v*/*v*) tetrachlorethylene (TCE)/heptane (density *ρ* = 1.3 g·cm^−3^), ultrasonicated on ice for 120 s with 15-s pulses and 15-s breaks. After filtration through a nylon mesh (20 μm pore size), the suspension was diluted three times with heptane and centrifuged (4 °C; 10 min; 3200× *g*). The pellet was resuspended in 2 mL 66:34 (*v*/*v*) TCE/heptane and the particles separated with a linear density gradient (25 mL, *ρ* = 1.43 − 1.62 g·cm^−3^) after 60 min centrifugation at 9000× *g* and 19 °C. From each gradient, six fractions (F1 to F6, 4.5 mL each) were collected from the top to the bottom using a peristaltic pump. Each fraction was then divided into six aliquots. To remove the solvents, the fractions were diluted three times with heptane and centrifuged (4 °C; 10 min; 3200× *g*). After elimination of the solvents, the six fractions were dried under air flux, at room temperature. Dry fractions were then stored at −20 °C before analysis.

### 4.3. Enzyme Activities

Aliquots of 7 to 10 mg dried leaf material were extracted in 500 µL as in [46]. Phosphoenolpyruvate carboxylase capacity was measured using a protocol derived from [47] in 50 mM Tricine-KOH (pH 7.8) containing 2.5 mM MgCl_2_, 0.2 mM NADH, 1 mM DTT, 10 mM NaHCO3, 3.33 unit.mL^−1^ NAD-malate dehydrogenase and 2.4 mM phosphoenolpyruvate. NADP-GAPDH was assayed in 0.1 M Tricine-KOH (pH 8) containing 6.25 mM ATP, 0.25 mM NADPH, 0.625 mM DTT, 12.5 unit·mL^−1^ phosphoglucokinase, 1.25 unit·mL^−1^ triose-phosphate isomerase and 4 mM 3-phosphoglycerate. Acid invertase and α-mannosidase were measured as in [48,49], respectively.

### 4.4. Metabolite Measurements

Free sugars, total chlorophylls and starch were extracted and measured as in [30]. Nitrate was measured in the ethanolic supernatant as described in [50]. Phosphorylated metabolites, nucleotide sugars, organic acids and amino acids were extracted from the dry fractions using boiling water as previously described by [51]. At the time of extraction 50 nmol of [U-^13^C_2_]-glycine, and 20 nmol of [U-^13^C_4_]-fumarate were added to each tube as internal standards (Sigma, Milwaukee, WI, USA). The extracts were lyophilized for 24 h, and then suspended in 500 µL of nanopure water at 4 °C and vortexed. The extracts were passed through a 3 kDa Amicon Ultra 0.5 mL filter device by centrifuging for 60 min at 4 °C and 14,000× *g* for the measurement of the amino acids, organic acids, phosphorylated compounds, and nucleotide sugars. These intracellular metabolites were separated and quantified using an Ultra High Performance Liquid Chromatography 1290 from Agilent Technologies (Agilent, Santa Clara, CA, USA) coupled to a hybrid triple quadrupole/ion mass spectrometer QTRAP 5500 from AB Sciex (AB Sciex Instruments, Framingham, MA, USA) as previously described by [51]. For the phosphorylated metabolites and organic acids, 40 µL of filtered extract was diluted in 160 µL of nanopure water, and 3 µL was analyzed by LC-MS/MS. Data were acquired and processed using Analyst 1.6.1 software package from AB Sciex.

The extraction and the analysis of the secondary metabolites were realized according to [52]. In details, the dry fractions were extracted with 1 mL methanol 70% blended for 1 min using an Ultra-thurrax and then centrifuged at 10,000× *g* for 10 min. The supernatant was transferred to a new tube and one additional mL was added to the pellet, vortexed and let for 2 h at room temperature. The mixture was centrifuged again at 10,000× *g* for 10 min and the recovered supernatant was mixed with the first one. The 2 mL solution was evaporated in a speed-vacuum until dryness. Then the dried pellet was dissolved in 500 μL methanol 70% and passed through a 0.22 μm filter. Extracts (1 μL) were analyzed on a Ultra-High Performance Liquid Chromatography (U-HPLC) system (Shimadzu) equipped with a photo Diode Array Detector (DAD) and a mass spectrometer. Samples were separated on a C18 kinetex (100 × 2.1 mm) column (Phenomenex). The mobile phase consisted in 0.1% formic acid in ultra-pure water (solvent A) and 0.1% formic acid in methanol (solvent B). The molecules were eluted through a gradient elution from 1 to 99% B for 13 min with a flow rate of 400 μL/min and then 3 min in 99% B. The column was then re-equilibrated to 1% B prior to the next run. Mass spectrometry analysis was carried out in ESI negative mode. Quantification was performed by measuring the area under each peak at 280, 320 or 350 nm, depending on the lambda max of each molecule, and expressed relative to calibration curves with chlorogenic acid (for caffeoyl hexaric acid isomers, caffeoyl-lysine and chlorogenic acid isomers), caffeoyl putrescine, rutin (for apio-rutin, rutin and kaempferol rutinoside). The tomatine concentration was determined on a 50-fold diluted sample according to [45].

### 4.5. Cell and Subcellular Volumes

To determine the volumes of the cell and of the subcellular compartments, micrographs were taken in the outer and in the inner part of three independent leaves. The volumes were measured as described previously [15] and then corrected from the shrinking effect due to the fixation [14,20]. Briefly, they estimated that the fixation reduces the total volume of the cell by a factor close to 63%, consequently to a drastic reduction of the volume of the vacuole (by 70%) and in a lesser extent of the other compartments (46%).

### 4.6. Data Analysis

PCA was performed using XLSTAT package (Addinsoft). The metabolite distribution within the three main compartments (cytosol, plastid and vacuole) was determined as described by [53] using a Python written program available upon request. The distribution of the subcellular compartments into the six fractions was determined using the mean distribution of several markers: ADP-glucose, ribulose-1,5-bisphosphate, chlorophyll and NADP-dependent GAPDH for the chloroplast; Sucrose-6-phosphate and phosphoenolpyruvate carboxylase for the cytosol; acid invertase or α-mannosidase, nitrate, CHA, CGA for the vacuole. It should be noted that nitrate and acid invertase were measurable only in leaf samples L5 and L12. As criteria for a best fit, we estimated the Manhattan distance, d_M_/2 = $(\sum_{i=1}^{6}\lceil x_{i}-y_{i}\rceil )/2$), *x_i_* and *y_i_* being the measured and the fitted percentages in fraction *i*, respectively. The fit was considered as “unexplained” (identified by the letter “U” in Table S2) when the average d_M_/2 was superior to 10% and that it exceeded 10% in at least 2 individual gradients [18]. For a given metabolite, if d_M_/2 was less than 10% for only 2 fractionations, the subcellular distribution was calculated based on these two samples only.

## 5. Conclusions

The present work aimed at studying the subcellular metabolome of tomato leaves as a function of their position on the plant and thus of their developmental stage. For this purpose, NAF, coupled with a cytology analysis was implemented. The results underlined the importance of using multiple markers for a robust analysis of the gradients during NAF. Specifically, metabolites such as phosphorylated compounds and phenolic acid esters, which can be assayed with better sensitivity than the enzymatic activities and are less prone to interferences, are novel markers highlighted in this study. On the other hand, the coupling with a cytology analysis allowed the determination of the concentrations at the subcellular level, underlining the quantitative importance of secondary metabolites in the composition of the vacuole of the youngest leaves. This work highlights the importance of studying the metabolome at the subcellular level, to reveal processes hidden by global analysis, in this case the reallocation of sucrose from the vacuole to the cytosol during leaf development. The measurement of subcellular concentrations is a real challenge for metabolomics. It is nonetheless essential for studying the regulation of metabolism since enzyme activities and therefore metabolic fluxes depend on local concentrations. We expect these approaches to be developed more intensively in the coming years to address these aspects of plant metabolism.

## Data Availability

The data presented in this study are openly available in Omics Dataverse (view at https://data.inrae.fr/dataverse/omics (accessed on 1 December 2021)) at https://doi.org/10.15454/LIGCQE (accessed on 1 December 2021).

## Supplementary Materials

The following are available online at https://www.mdpi.com/article/10.3390/metabo11120848/s1, Figure S1: Fractional volumes of chloroplast, cytosol and vacuole according to the leaf position, Figure S2: Principal component analysis of metabolites, depending on the period of the day, Figure S3: Evolution of metabolite contents according to the leaf position, Figure S4: Distribution of the subcellular markers in non-aqueous gradients of tomato leaf, Figure S5. Comparison between the tri- and a two-compartmented model for the determination of the vacuolar percentage of sugars, organic acids and secondary metabolites. Figure S6: Enzyme activities in tomato plant leaves, depending on the leaf position title, Figure S7: Subcellular distribution of the major amino acids in tomato leaves harvested at 4 positions on the plant, Figure S8: Subcellular distribution of the major phosphorylated compounds plus pyruvate in tomato leaves harvested at 4 positions on the plant, Figure S9: Cellular and subcellular sugar ratios according to the age of the leaf, Figure S10: Vacuolar and cytoplasmic concentrations of the major amino acids and secondary metabolites in tomato leaves harvested at 4 positions on the plant. Figure S11: Composition of the vacuole depending on the position of the leaf on the plant, Table S1: Experimental data and statistical analysis for metabolite contents and enzyme activities, Table S2: Non-aqueous fractionation results and estimation of metabolite concentrations.
