# Supplementary material for: The Evolution of Leaf Function during Development Is Reflected in Profound Changes in the Metabolic Composition of the Vacuole
Source: Metabolites. 2021 Dec 6;11(12):848. doi: 10.3390/metabo11120848 (PMC8707551; doi:10.3390/metabo11120848)
Supplement: Supplementary file 1 [file metabolites-11-00848-s001.zip › supdata_metabolites/Sup Fig.pptx]

## Slide 1
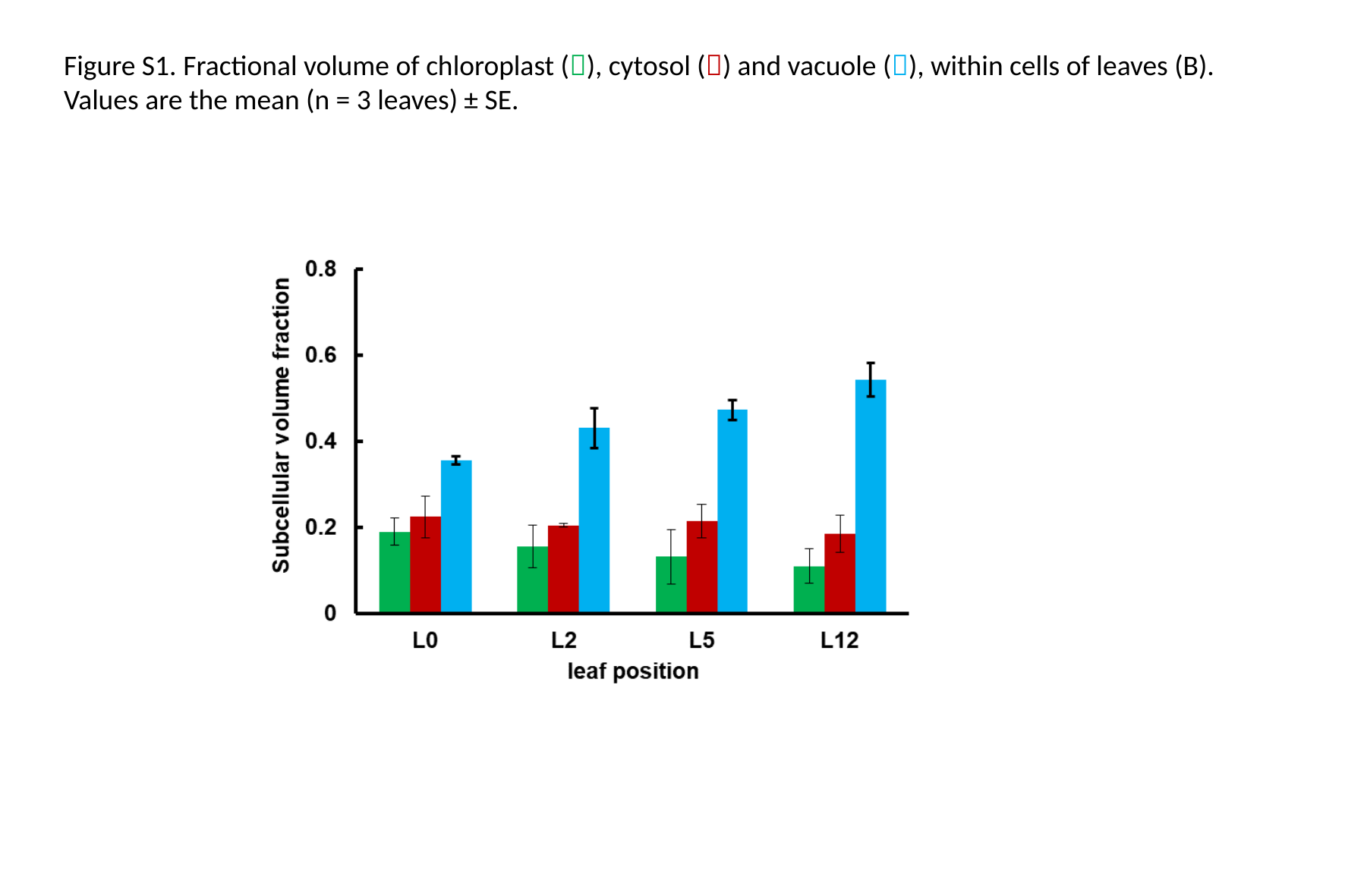

Figure S1. Fractional volume of chloroplast (), cytosol () and vacuole (), within cells of leaves (B).
Values are the mean (n = 3 leaves) ± SE.

## Slide 2
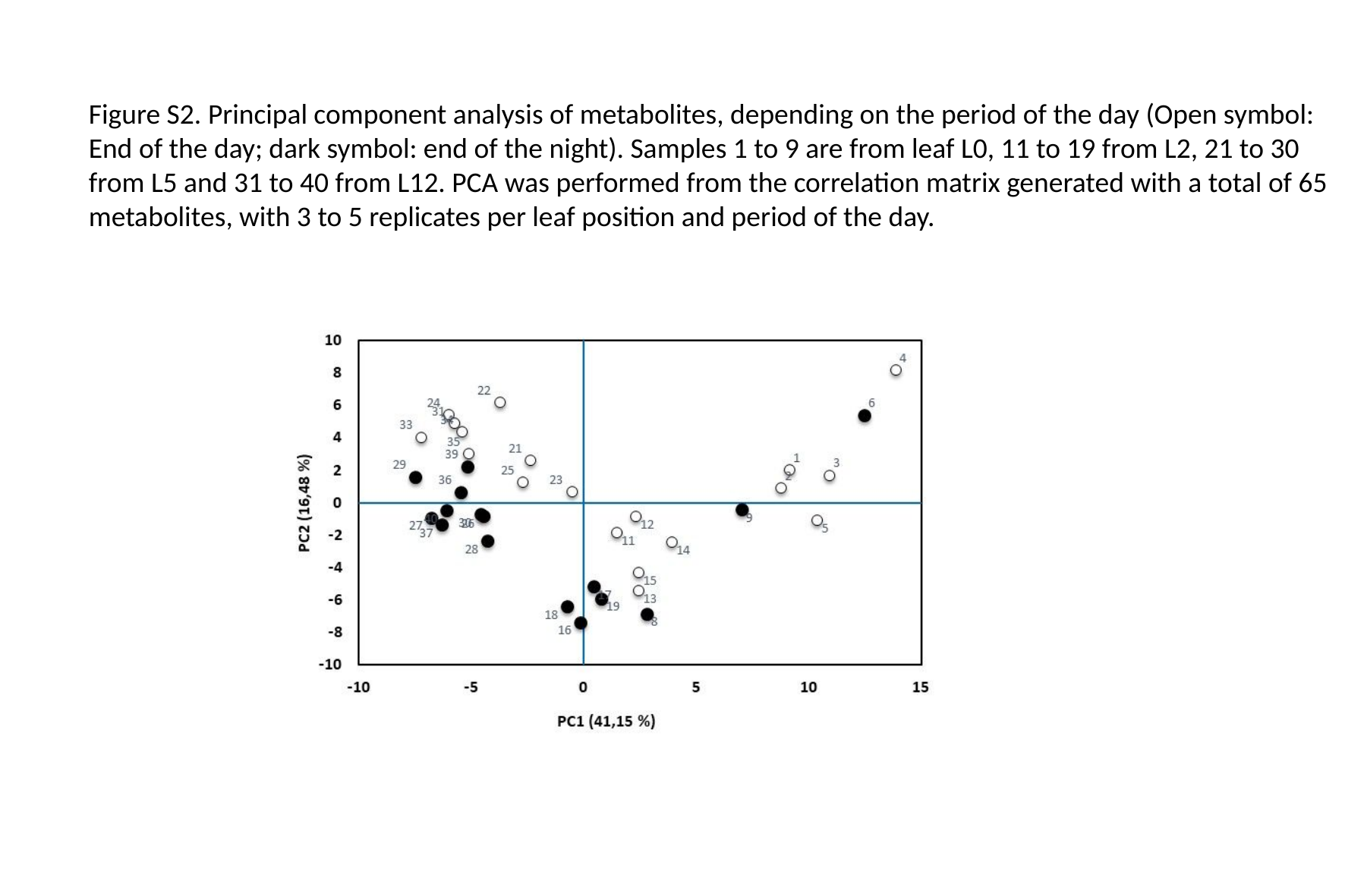

Figure S2. Principal component analysis of metabolites, depending on the period of the day (Open symbol: End of the day; dark symbol: end of the night). Samples 1 to 9 are from leaf L0, 11 to 19 from L2, 21 to 30 from L5 and 31 to 40 from L12. PCA was performed from the correlation matrix generated with a total of 65 metabolites, with 3 to 5 replicates per leaf position and period of the day.

## Slide 3
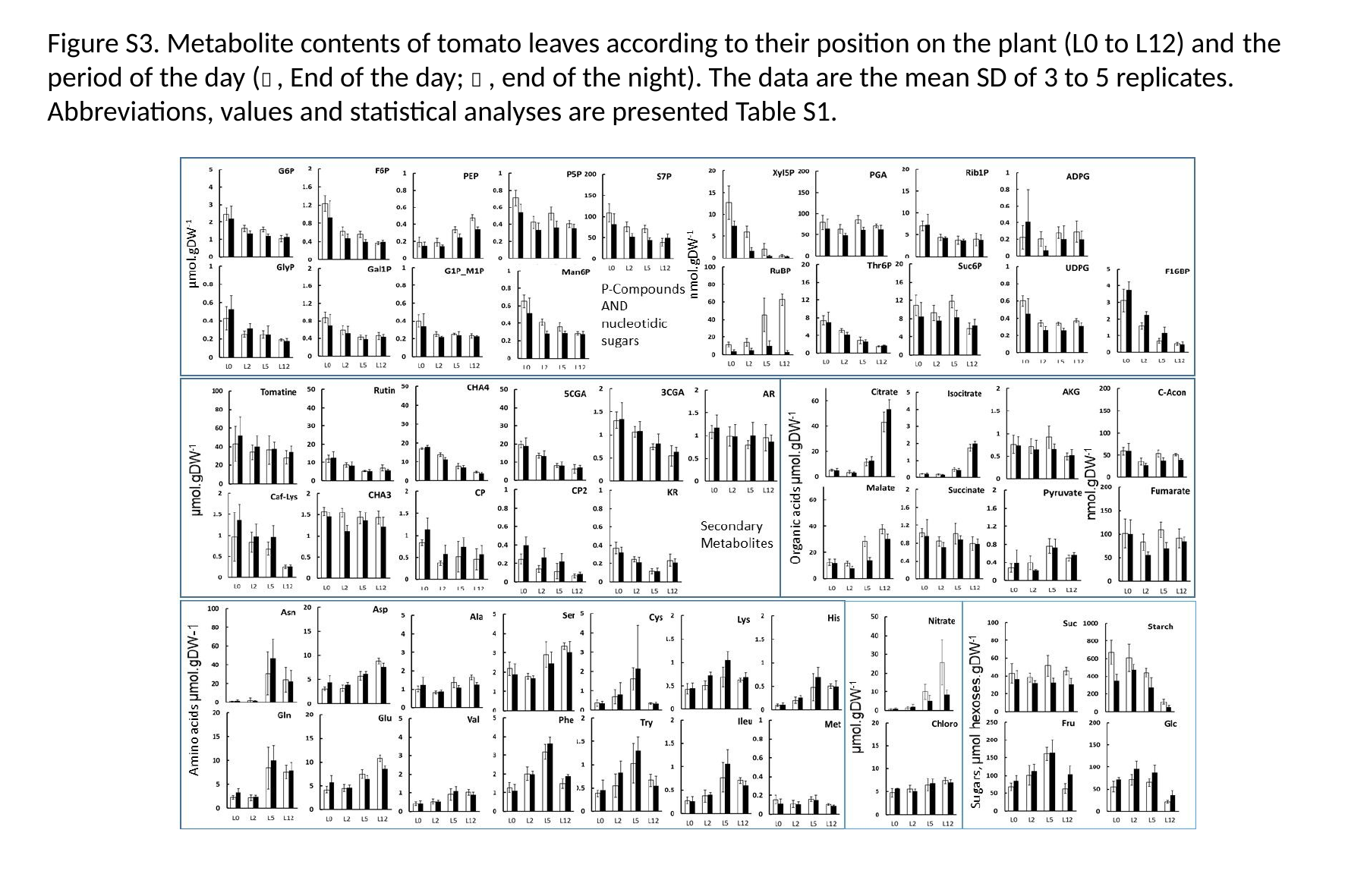

Figure S3. Metabolite contents of tomato leaves according to their position on the plant (L0 to L12) and the period of the day ( , End of the day; γ , end of the night). The data are the mean SD of 3 to 5 replicates. Abbreviations, values and statistical analyses are presented Table S1.

## Slide 4
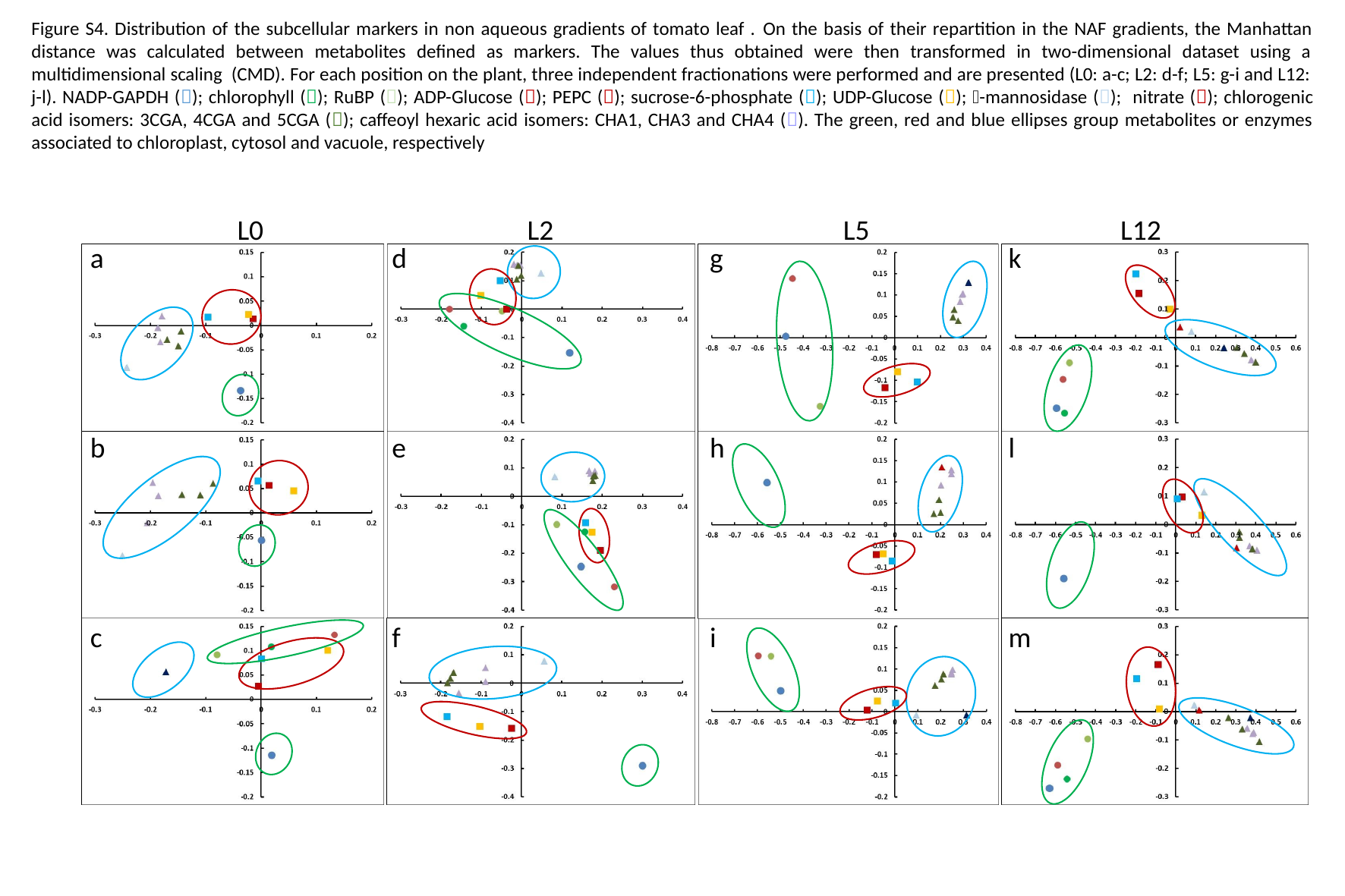

Figure S4. Distribution of the subcellular markers in non aqueous gradients of tomato leaf . On the basis of their repartition in the NAF gradients, the Manhattan distance was calculated between metabolites defined as markers. The values thus obtained were then transformed in two-dimensional dataset using a multidimensional scaling (CMD). For each position on the plant, three independent fractionations were performed and are presented (L0: a-c; L2: d-f; L5: g-i and L12: j-l). NADP-GAPDH (); chlorophyll (); RuBP (); ADP-Glucose (); PEPC (); sucrose-6-phosphate (); UDP-Glucose (); α-mannosidase (); nitrate (); chlorogenic acid isomers: 3CGA, 4CGA and 5CGA (); caffeoyl hexaric acid isomers: CHA1, CHA3 and CHA4 (). The green, red and blue ellipses group metabolites or enzymes associated to chloroplast, cytosol and vacuole, respectively
L0 L2 L5 L12
k
l
m
a
b
c
g
h
i
d
e
f

## Slide 5
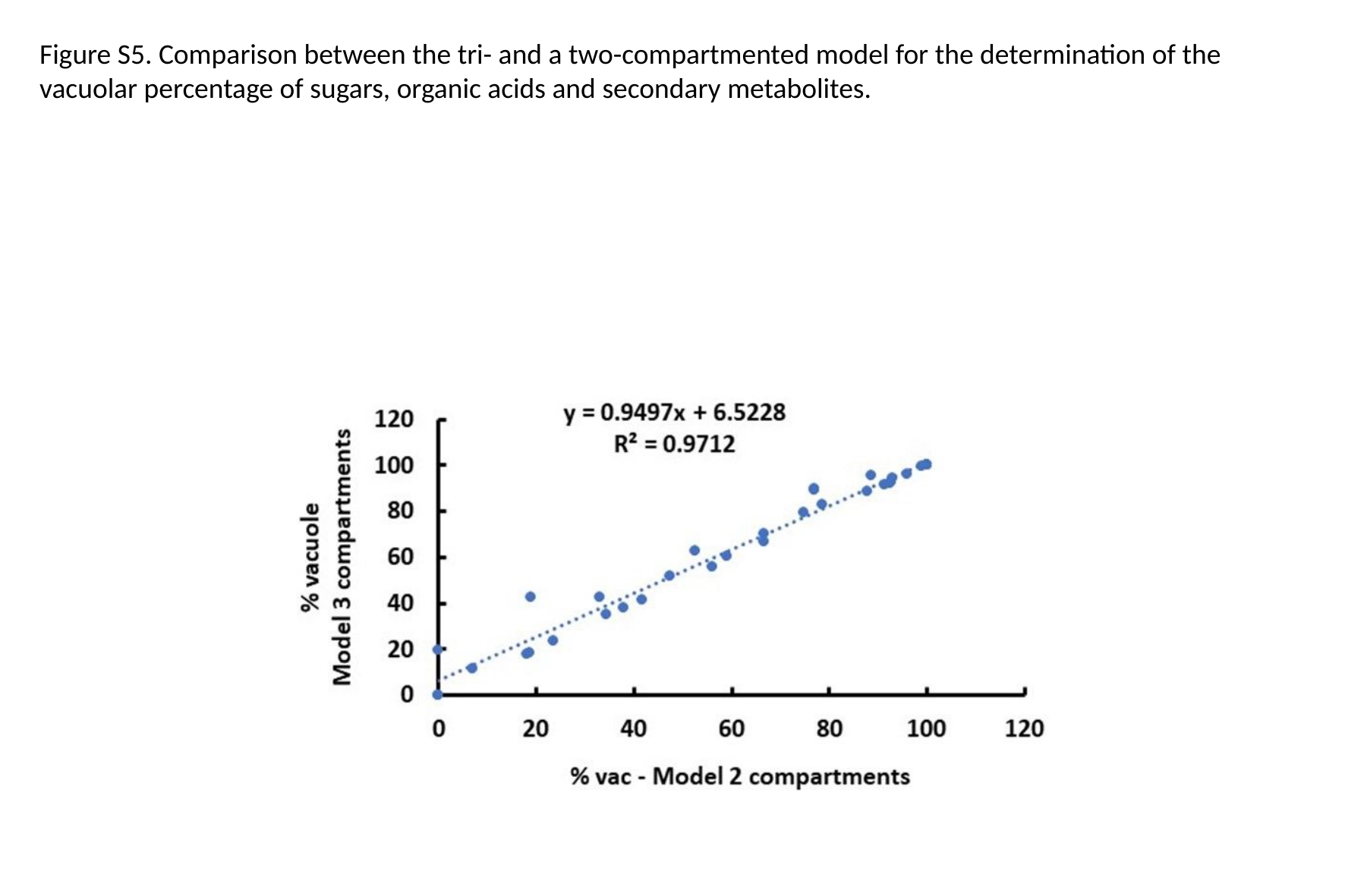

Figure S5. Comparison between the tri- and a two-compartmented model for the determination of the vacuolar percentage of sugars, organic acids and secondary metabolites.

## Slide 6
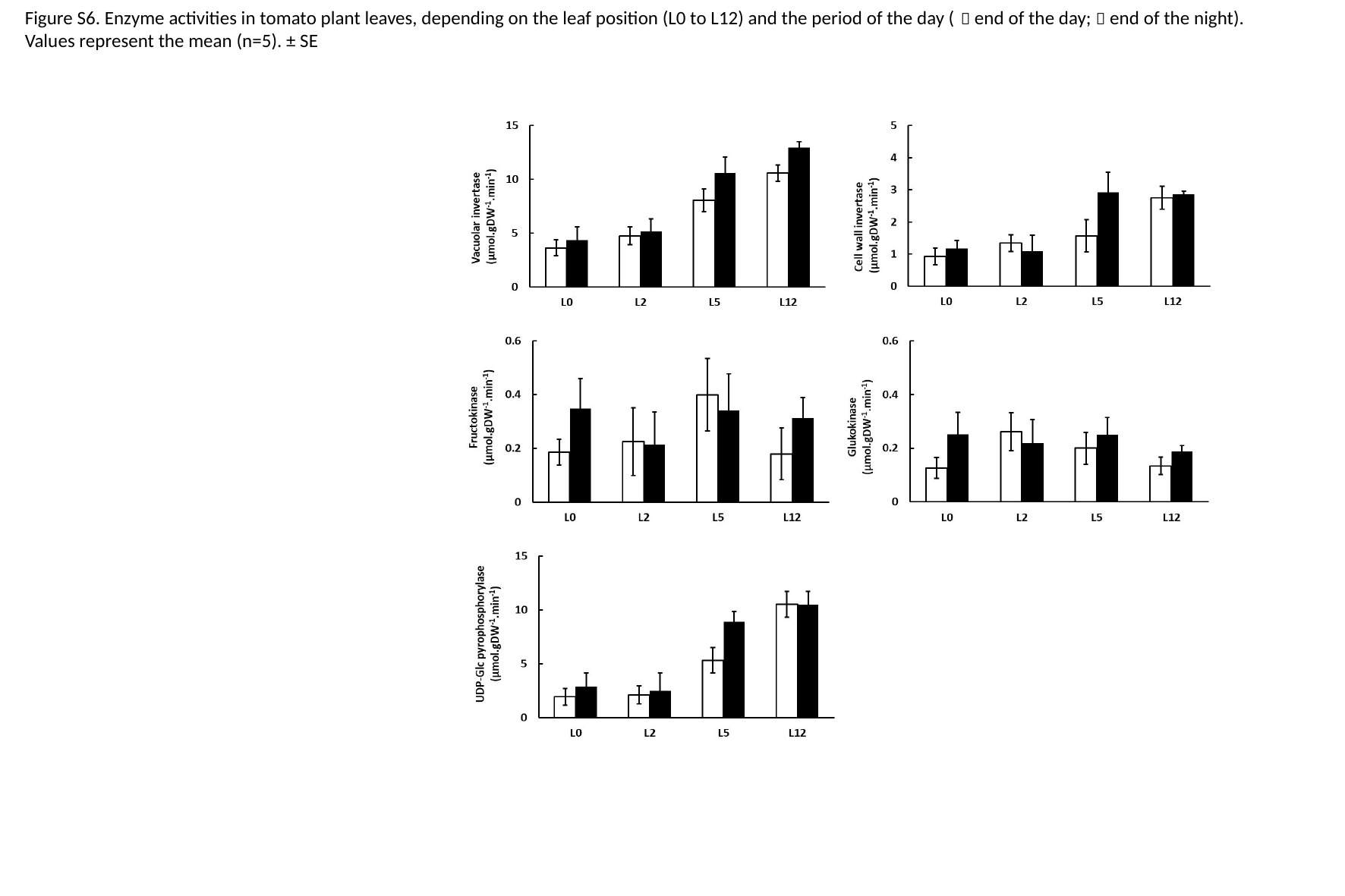

Figure S6. Enzyme activities in tomato plant leaves, depending on the leaf position (L0 to L12) and the period of the day (  end of the day; γ end of the night). Values represent the mean (n=5). ± SE

## Slide 7
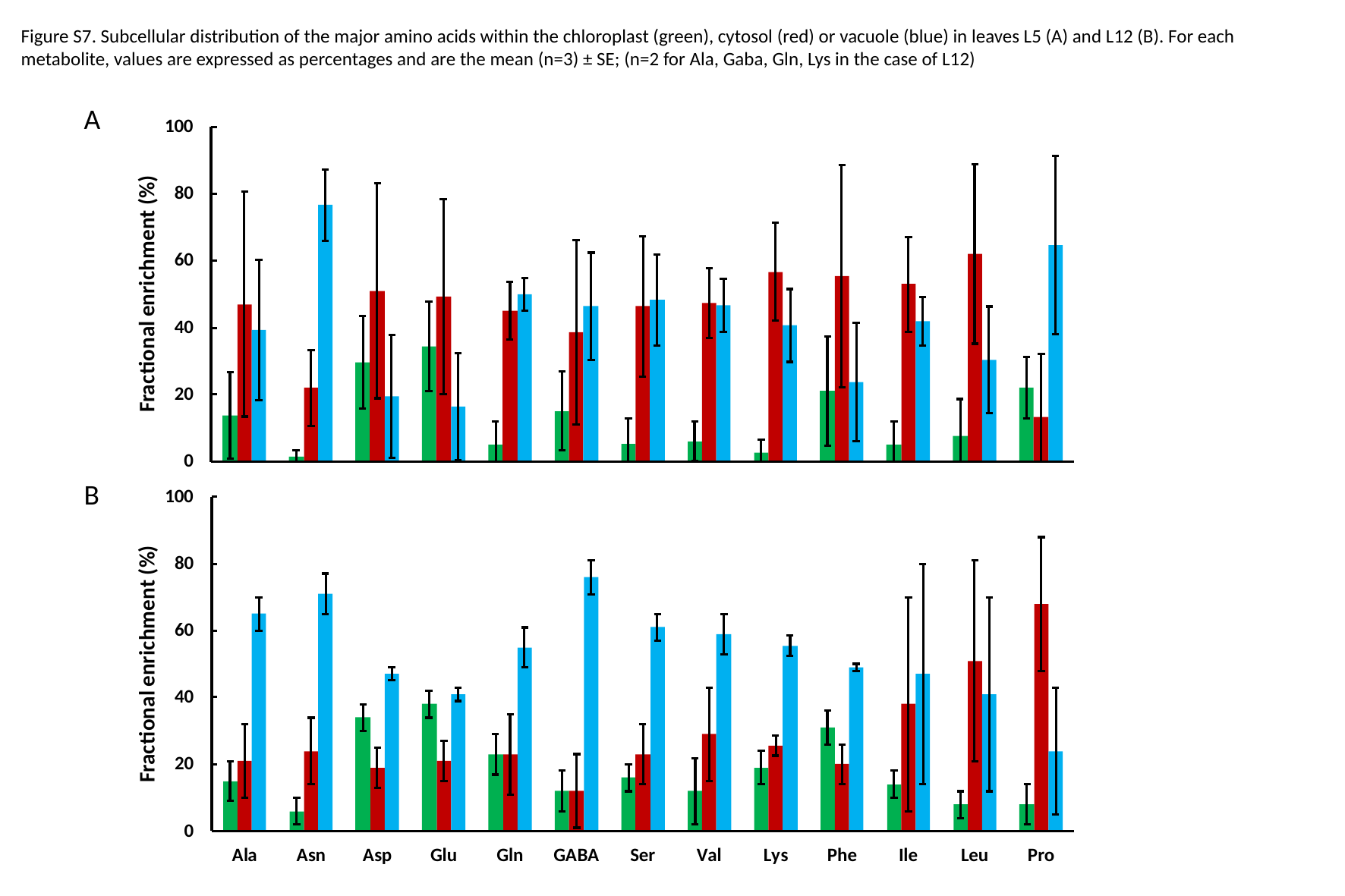

Figure S7. Subcellular distribution of the major amino acids within the chloroplast (green), cytosol (red) or vacuole (blue) in leaves L5 (A) and L12 (B). For each metabolite, values are expressed as percentages and are the mean (n=3) ± SE; (n=2 for Ala, Gaba, Gln, Lys in the case of L12)
A
B

## Slide 8
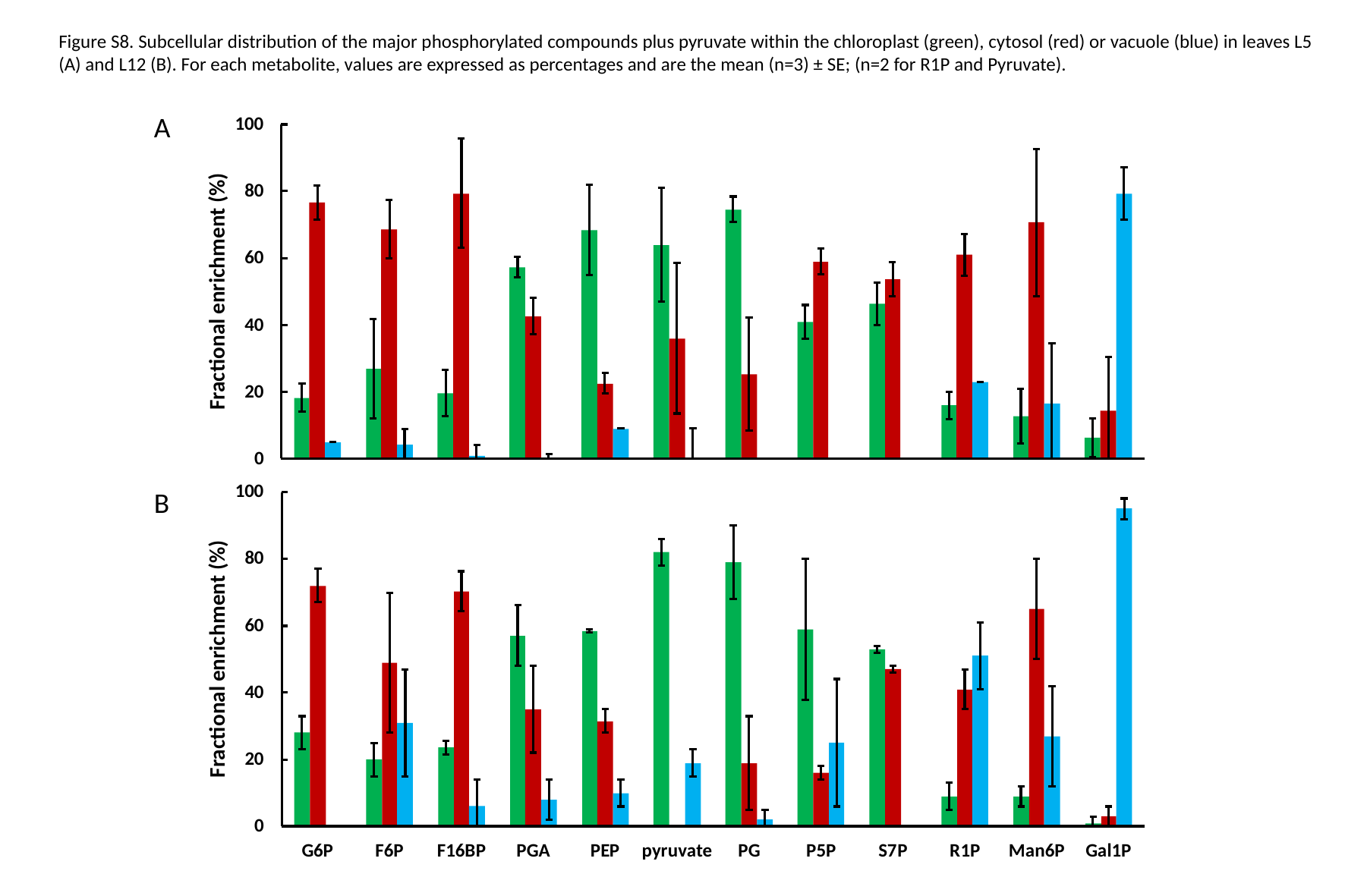

Figure S8. Subcellular distribution of the major phosphorylated compounds plus pyruvate within the chloroplast (green), cytosol (red) or vacuole (blue) in leaves L5 (A) and L12 (B). For each metabolite, values are expressed as percentages and are the mean (n=3) ± SE; (n=2 for R1P and Pyruvate).
A
B

## Slide 9
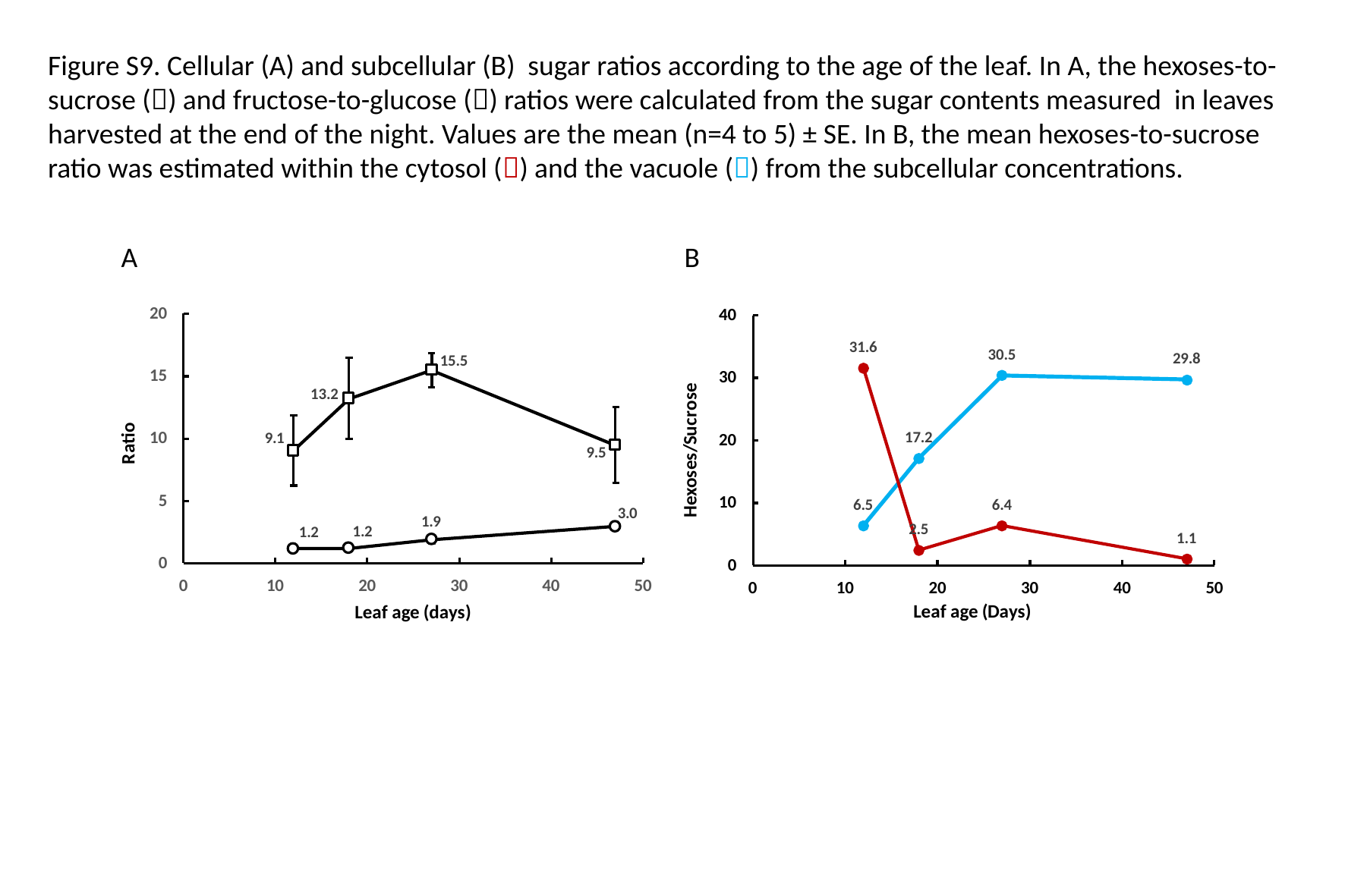

Figure S9. Cellular (A) and subcellular (B) sugar ratios according to the age of the leaf. In A, the hexoses-to-sucrose () and fructose-to-glucose () ratios were calculated from the sugar contents measured in leaves harvested at the end of the night. Values are the mean (n=4 to 5) ± SE. In B, the mean hexoses-to-sucrose ratio was estimated within the cytosol () and the vacuole () from the subcellular concentrations.
A B

## Slide 10
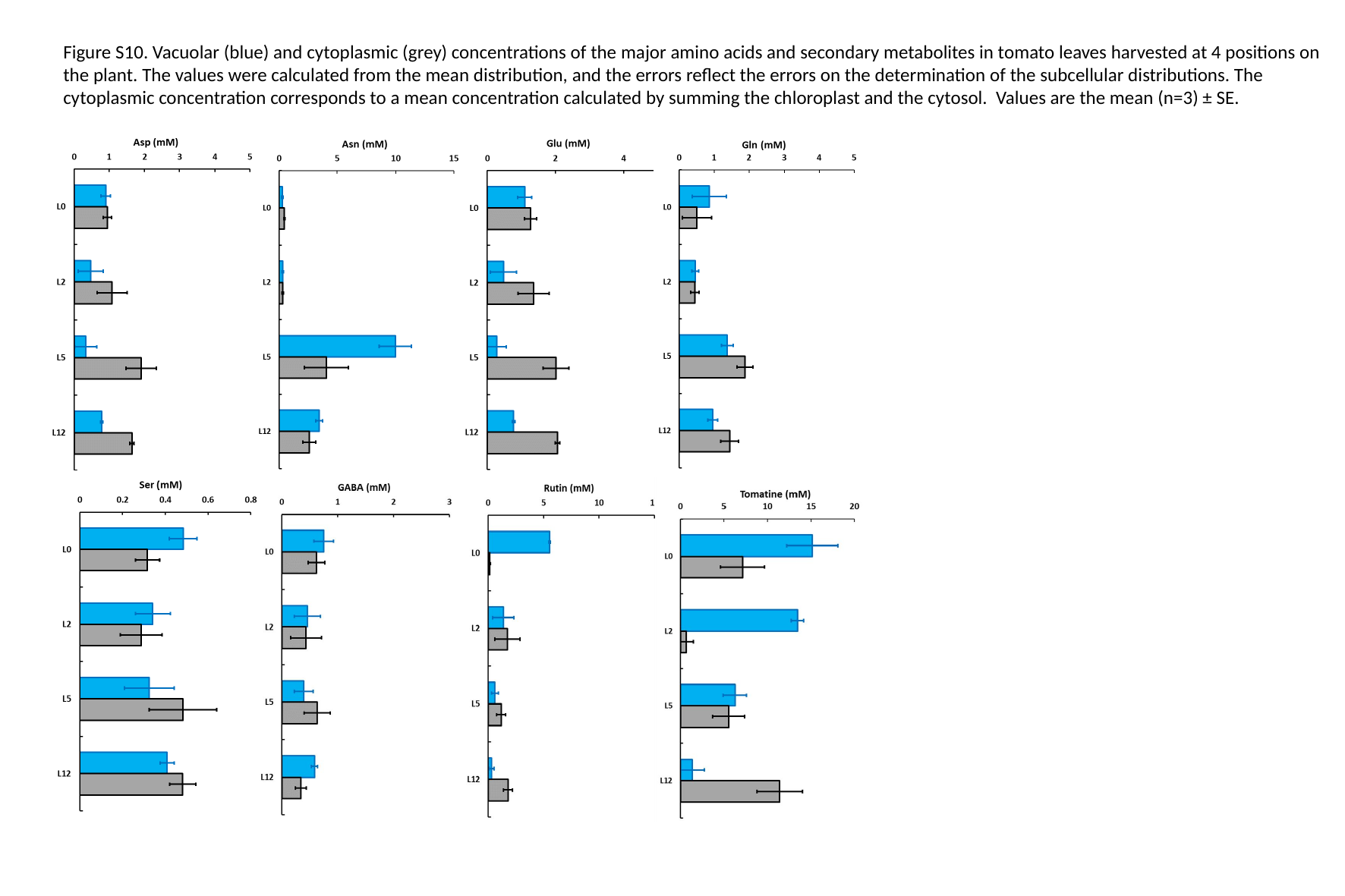

Figure S10. Vacuolar (blue) and cytoplasmic (grey) concentrations of the major amino acids and secondary metabolites in tomato leaves harvested at 4 positions on the plant. The values were calculated from the mean distribution, and the errors reflect the errors on the determination of the subcellular distributions. The cytoplasmic concentration corresponds to a mean concentration calculated by summing the chloroplast and the cytosol. Values are the mean (n=3) ± SE.

## Slide 11
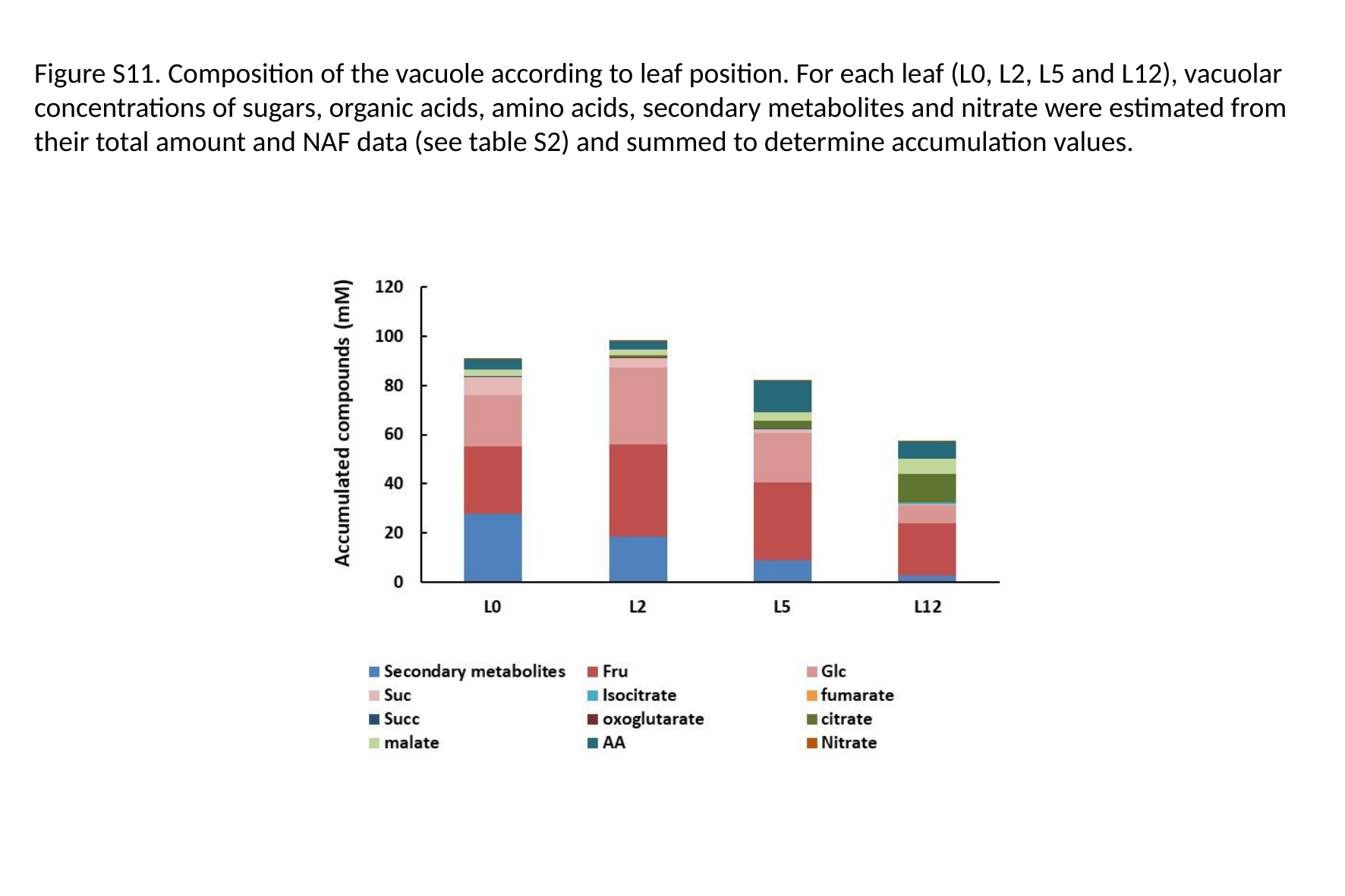

Figure S11. Composition of the vacuole according to leaf position. For each leaf (L0, L2, L5 and L12), vacuolar concentrations of sugars, organic acids, amino acids, secondary metabolites and nitrate were estimated from their total amount and NAF data (see table S2) and summed to determine accumulation values.
